# A participant perspective on collaborative reflection: video-stimulated interviews show what residents value and why

**DOI:** 10.1007/s10459-020-10026-7

**Published:** 2021-02-15

**Authors:** Marije van Braak, Esther Giroldi, Mike Huiskes, Agnes D. Diemers, Mario Veen, Pieter van den Berg

**Affiliations:** 1 Erasmus Medical Center, Dr. Molewaterplein 40, P.O. Box 2040, 3015 GD Rotterdam, The Netherlands; 2 Maastricht University, Maastricht, The Netherlands; 3 Rijksuniversiteit Groningen, Groningen, The Netherlands; 4 University Medical Center Groningen, Groningen, The Netherlands

**Keywords:** Collaborative reflection, Participant perspective, Video-stimulated interview, Medical education

## Abstract

The potential of reflection for learning and development is broadly accepted across the medical curriculum. Our understanding of how exactly reflection yields its educational promise, however, is limited to broad hints at the relation between reflection and learning. Yet, such understanding is essential to the (re)design of reflection education for learning and development. In this qualitative study, we used participants’ video-stimulated comments on actual practice to identify features that do or do not make collaborative reflection valuable to participants. In doing so, we focus on aspects of the interactional process that constitute the educational activity of reflection. To identify valuable and less valuable features of collaborative reflection, we conducted one-on-one video-stimulated interviews with Dutch general practice residents about collaborative reflection sessions in their training program. Residents were invited to comment on any aspect of the session that they did or did not value. We synthesized all positively and negatively valued features and associated explanations put forward in residents’ narratives into shared normative orientations about collaborative reflection: what are the shared norms that residents display in telling about positive and negative experiences with collaborative reflection? These normative orientations display residents’ views on the aim of collaborative reflection (educational value for all) and the norms that allegedly contribute to realizing this aim (inclusivity and diversity, safety, and efficiency). These norms are also reflected in specific educational activities that ostensibly contribute to educational value. As such, the current synthesis of normative orientations displayed in residents’ narratives about valuable and less valuable elements of collaborative reflection deepen our understanding of reflection and its supposed connection with educational outcomes. Moreover, the current empirical endeavor illustrates the value of video-stimulated interviews as a tool to value features of educational processes for future educational enhancements.

## Introduction

Reflection education plays a key part in medical curricula of all sorts: from basic medical training to medical specialist training to continuous medical education for accomplished professionals (Hellermann 2009; Sandars 2009). Reflective activities in medical education take their importance from the assumption that reflection fosters learning, which renders competent professional behavior (Aronson 2011; Sandars 2009; Schei et al. 2019; Wilson 2020). Yet, this assumption is not consistently buttressed with empirical evidence: the efficacy of reflection for learning and professional development varies between studies and contexts (Sandars 2009; Uygur et al. 2019). Evidence for long-term positive effects on professional development is limited (Mann et al. 2009; Sandars 2009), but reflection has been shown to increase learning and professional development in the shorter term (Sandars 2009) and in specific contexts, such as complex patient cases (Mann et al. 2009; Sandars 2009).

In this paper, we describe *in participant terms* the educational value of particular features of the interactional process which constitutes the educational activity of collaborative reflection. Participant perspectives on the value of educational activities that are supposed to facilitate or foster reflection have been described as a valuable resource for understanding “how reflective learning within the curriculum can be better developed to increase engagement from learners” (Vivekananda-Schmidt et al. 2011, p. 1). To date, however, reports of what participants value in collaborative reflection are still uncommon. Studies describing participant perspectives mainly focus on students’ perceptions of the *effect* of reflection on learning and development, not the *mechanism* that explains the relation. In research across the medical curriculum, students report that written reflection exercises improve their skills to formulate learning needs, integrate knowledge from different sources (Grant et al. 2006), and learn from experience (Larsen et al. 2016). Also, these exercises allegedly raised awareness of the students’ learning (Larsen et al. 2016), boosted their confidence about already present knowledge and skills (Grant et al. 2006), and provided support and encouragement (Özçakar et al. 2009). As for peer reflection sessions, these have been reported to train students’ skills in challenging and supporting others’ views, improve their readiness for practice (Green 2002), reduce stress, improve patient care, and stimulate professional development (Lutz et al. 2013). Reflective activities are generally rated positively, but some researchers have reported students’ evaluation of reflection as an unnecessary burden (cf. Vivekananda-Schmidt et al. 2011; Murdoch-Eaton and Sandars 2014; Veen et al. 2020). In summary, participants appear to value reflection for its various effects on learning outcomes, but are also critical of the investment required to achieve that value.

Findings on the perceived effects of reflection illuminate its potential benefits and pitfalls for learning and development. Yet, they shed no light on the mechanisms that explain *why* reflection contributes to learning. Other than data on general characteristics of reflective activities that appear to be valued (e.g., peer support in collaborative reflection sessions (Chou et al. 2011) and facilitation of reflective processes (McEvoy et al. 2016), we lack empirical data on the actual mechanisms that lend reflection its educational promise. Yet, those mechanisms are crucial in determining what works for whom and in which circumstances (Giroldi et al. 2014; Wong et al. 2012). This knowledge is the cornerstone of medical curricula to promote reflection and of teacher training to facilitate reflection. In our study, therefore, we explore participants’ views on the value of an educational activity of which the aim is to collaboratively reflect on professional practice (van Braak et al. submitted). We focus particularly on their views about the *mechanisms* that explain why certain aspects of the activity do or do not create educational value.

## Methods

### Data collection

We conducted video-stimulated interviews with residents participating in 24 recorded collaborative reflection sessions from all eight general practitioner (GP) training institutions in The Netherlands. During weekly sessions scheduled throughout their three-year GP training program, small groups of 5–15 GP residents collaboratively discuss experiences from practice (Veen and De la Croix 2017). The sessions typically last 1–1.5 h and are facilitated by one or two teachers (an experienced GP and/or a behavioral scientist or psychologist), whose task is to facilitate reflection for professional learning and development. This type of collaborative reflection sessions originates from Balint group meetings, during which professionals “explore difficult interactions with patients through case presentations and discussions” which “broaden their perspective on the initial difficulty they experienced, and can influence their overall perception of their practice and interactions with patients” (Van Roy et al. 2015, p. 686; Balint 1955).

We selected sessions for recording using maximum variation sampling over the eight Dutch GP vocational training institutes and year of GP training program (see Table 1). All residents and teachers of the recorded groups gave written informed consent. On the informed consent form, residents could agree to do a video-stimulated interview and, eventually, 31 residents were interviewed within two weeks of the recording (see Table 1).

**Table 1:** Overview of recorded groups and interviews conducted per year/institute

	Year 1	Year 2	Year 3	Total
Institute A	x (1) x (1)	x (1) x (1) x (1)	x (1) x (1)	7 (7)
Institute B	x (2) x (1) x (2)	x (1) x (1)	x (2)	6 (9)
Institute C	x (2)	x (2)	x (1)	3 (5)
Institute D	x (1)	x (2)	x (1)	3 (4)
Institute E	x (1) x (1)			2 (2)
Institute F			x (2)	1 (2)
Institute G	x (1)			1 (1)
Institute H		x (1)		1 (1)
Total	10 (13)	8 (10)	6 (8)	24 (31)

NB. Each recording is denoted by an x, followed by the number of interviews about that recording

Interviews were conducted between May 2017 and January 2019 by two authors (EG and MB) who were not involved in the design or teaching of collaborative reflection sessions, giving them a relatively neutral stance to the educational activity. As anticipated, their ‘outsider’ role created a safe environment for residents to express their potentially critical opinions of the recorded sessions. Interviews followed a pilot-tested interview protocol (cf. van Braak et al. 2018). Participants gave written informed consent prior to the interview. During the 45–60 min. interview, residents were asked to select for reflection a part of the recorded session that was in any respect noteworthy for them. The interviewer instructed the resident to comment on any aspect of the viewed recording that they had experienced as positive or negative. Residents were encouraged to stop the recording and start talking whenever they wished; they were prompted only minimally (van Braak et al. 2018) to minimize researcher influence on what was evaluated. Interviews were audio-recorded for transcription, during which recognizable personal and institutional information was anonymized. Ethical approval for this study was obtained from the Ethical Review Board of the Dutch Association of Medical Education (NVMO), dossier 829.

### Analysis

Residents’ narratives (Gee 2014) elicited in the interviews were analyzed by MB, MV, and EG using Template Analysis (King 2012) in Atlas.ti. Template Analysis is a thematic coding approach that—other than, for example, grounded theory—allows researchers to take a “contextual constructivist stance that is sceptical of the existence of ‘real’ internal states to be discovered through empirical research” (King 2012, p. 418). This affordance, as well as its flexibility in developing a coding structure based on a priori and deductively established codes (i.e., the template), particularly suits our research aims.

EG and MB first pilot coded one interview to decide the unit of analysis and get a feel for a possibly useful coding template. They decided to proceed coding by identifying all interview fragments in which participants displayed a norm about an aspect of the reflective discussion interaction (i.e., reflected on the value or lack of value of that aspect). Each identified interview fragment would be coded using three coding categories established a priori: the object (what is seen as valuable or not valuable), its valence (whether it was seen as valuable, not valuable, or probably ambiguous), and the mechanism (why the object would contribute to educational value or not). These categories constitute the basic structure of the coding template, which could then be flexibly applied to the remaining interviews. MB coded all remaining interviews. MV double coded every fifth interview, after which MB and MV conferred for consensus; codes in already coded interviews were adapted accordingly.

Following this initial coding round, MB merged the overlapping codes and organized the resulting codes into central themes (e.g. structure, safety) while preserving the connections interviewees had made between positively and negatively valued aspects and mechanisms perceived to account for this value or lack of value. Building on the central themes, MB and MH then identified the norms underlying the ascription of value or lack of value to particular aspects of discussion. The findings presented below are a synthesis of these shared normative orientations (Maynard and Heritage 2005)—“normative rules” that “both define what immediate ends should and should not be sought, and limit the choice of means to them in terms other than those of efficiency” (Parsons, as cited in Hamilton 1985, p. 62).

## Results

In the interviews, residents discuss valuable collaborative reflection sessions in terms of providing *educational value for all*. In the residents’ discourse, *inclusivity* *and* *diversity*, *safety*, and *efficiency* are key norms that are perceived to contribute to the sessions’ main goal of educational value for all. In the following, we first elaborate on that goal, then discuss the normative orientations that supposedly contribute to it. Finally, we present the residents’ views on the value of activities and contributions to ongoing reflective interaction in light of the normative orientations. See Table 2 for a summary of the findings.

**Table 2:** Summary of the main findings: participants’ perspectives on collaborative reflection sessions

*Main aim* is to achieve educational value for all	
*Norms* that are perceived conditional to creating educational value for all	1. Inclusivity and diversity
	2. Safety
	3. Efficiency
*Activities that contribute to these norms (per phase)*	*Telling*: create telling space; share tellable and discussable stories
	*Exploration*: structuring to focus on main issue in telling; room to ‘feel out’; diversity of questions
	*Discussion*: dive deeper into potential causes, explore possible directions, hint at solutions; leave room for group process; monitor and jump in as expert when needed
	*Conclusion*: summarize uptake/‘learnable’

### Collaborative reflection: aim

**Fig. 1:**
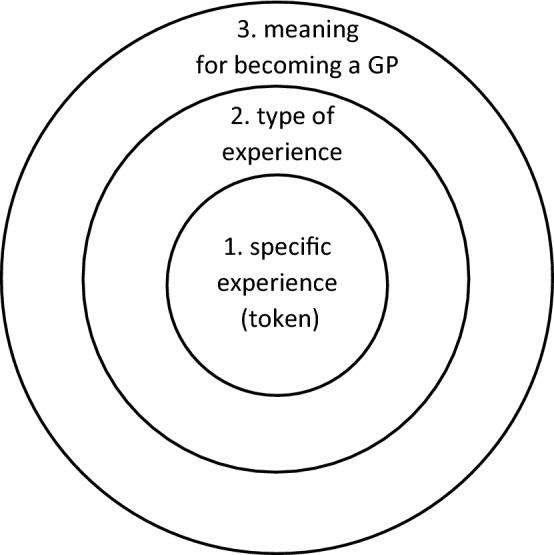
Graphic representation of concentric circles of value derived from case discussions in the collaborative reflection setting

Residents consistently addressed a common benchmark for good collaborative reflection: “educational value for everyone” (interview F803). This value is represented as a ‘layered’ value, constructed throughout the reflective discussion in three concentric circles (Fig. 1).

Building on a *specific experience* shared by one individual (circle 1), the group should treat the experience as a token of a *type of experience* (circle 2) that is recognizable as a relevant and *meaningful* issue that carries a sense of urgency *in the process of becoming a GP* (circle 3). For example, a resident may share an experience of a difficult patient contact (circle 1), which is treated as a token of a broader interactional dilemma such as discussing a difficult matter with a patient while not damaging the relation of trust with the patient (circle 2). This is ultimately discussed in the context of being a GP, who has to be able to say things that either would not be said or would be very delicate to express in daily life (circle 3). This token-type relation allows for educational interaction that serves both the individual who experienced the situation as well as others who might have had or will experience similar situations. Talking about what happened may seem a tedious practice at first and a long shot toward professional development, but it is perceived as carrying a significance that highlights the unique quality of the participants’ current situation in training: “a luxury position that you won’t have once you’ve graduated, and […] this is the time to use it” (D700). Ideal collaborative reflection discussion, thus, is relevant for the practice of multiple participants beyond the here and now.

Though the importance of achieving educational value is widely shared, the interviews display residents’ disagreement about the nature of this value. Some appreciate the value of obtaining new knowledge, a solution to a problem or advice about an issue. Given their comparable situations, residents can relate to each other’s issues, which increases the perceived value of their advice. Others, though, regard many discussions as “too solution-oriented” (C811). They value the significance of recognition by “peers who are in the same boat” (D753). Its relativizing and reassuring potential, in their view, might benefit long-term practice more than solutions or advice do. For some, sharing is already valuable enough as an activity in itself. It helps to organize one’s thoughts or just “get things off your mind” (D753) with the group merely functioning as a sounding board. This is one of the main points in which residents’ views diverge: should collaborative reflection discussion carry value beyond the sharing? Mostly, yes. As one resident put it:

“I don’t really like it when it’s just venting for the sake of venting. […] I really think it should produce, you know, a return on learning, that you get something out of it” (D753).

Another view that residents consistently express is that it is not enough for the reflective discussion to *have educational value,* but that value should also apply to *everyone* present. Summarizing a session they attended, one resident commented on its value for the group members:

Yes, for [name of one resident] personally, I think it had [value], but for the group, I thought, it wasn’t the most clarifying of sessions. Last week’s session was, I thought, far better because [then] many more people brought up their personal issues (E821).

The resident quoted here distinguishes personal benefit from group benefit, characterizing the limited value as a lack of clarification. In contrast to the session currently discussed, last week’s session featured many more people’s personal input—which supposedly contributed to its educational value.

### Collaborative reflection: norms

To realize *educational value for all*, collaborative reflection interaction should, according to the residents, be *inclusive and diverse*, *safe*, and *efficient.*

#### Inclusivity and diversity

In residents’ talk about the collaborative reflection sessions, the bottom line for creating educational value is for something *to be brought up* for discussion. If issues go unshared, stories remain untold, responses are withheld, turns are passed, what can be learned? Residents orient to a norm of inclusive participation: everyone should get the chance to bring something up for discussion and contribute to the discussion of what is brought up. Only in that way is value created for all, as one resident explained:

Sometimes I’m rather passive, because then I think, well, I just can’t do it. I won’t yell over other people’s voices. Um, yeah, it differs quite a lot, actually. Some days I’ll do my [best]. Some days I’ll find my story really important and then I’ll stand up for [myself]. Then I’ll always try to speak up. But, um, yeah, I think that […] sometimes I find it hard to find the space for that. Mostly it’s the same people […] who probably benefit more from the exchange [of experiences] because they have more turns (C808).

Standing up for one’s right to have a turn, as this resident puts it, may be one way of obtaining a turn, but residents also value the shared responsibility of all participants (including teachers) to distribute turns fairly. Both overtly active and apparently passive participants should learn to dose their participation in the group discussion. A variety of participants creates a diversity of perspectives, which the interviewees evaluated as beneficial to the learning process. Importantly, though, residents do not like being forced to participate, as compulsory contribution may reduce authenticity and compromise a safe learning environment, which in turn depreciates the educational value.

#### Safety

Related to the norm of inclusivity and diversity is residents’ orientation to ‘safety’, that is “feeling safe [enough] to bring up something for discussion”, “to not turn on each other”, “to be able to say things to each other respectfully, even the less pleasant things” (B870). Participants regard a safe learning environment as one that allows non-judgmental interaction that encourages vulnerability and openness. In such an environment, everyone respects each other, including possibly opposing, idealized, unorthodox views and whatever situation they are in. Creating a safe learning environment, many residents comment, is a co-construction of teachers and residents. Residents see it as the task of the *teacher* to treat mistakes as learning opportunities, not as evidence for low assessment. *Residents* can contribute to a safe climate by welcoming others’ viewpoints and opening up about personal issues relevant to becoming a GP. Teachers can validate such displays of vulnerability by complimenting residents who do so for the example they set for others in the group.

#### Efficiency

Inclusivity, diversity, and safety could be interpreted as a wildcard for long and deep reflection sessions. Residents, however, stress the importance of efficient discussion. Probably in parallel with their professional practice, they appreciate interactional behavior that promotes progression toward the educational end in terms of pace and ‘depth’ of discussion. Such progression requires structured yet dynamic interaction, which is mostly perceived as the teachers’ responsibility. Teachers’ contributions are weighed for their potential to spur discussion to higher levels and time-efficient processes. One resident, for example, rated a certain teacher’s “intervention” (raising a new subtopic) as “a very good contribution” (A823) because it smoothed the interactional process and reopened the discussion about an issue that was relevant both to the case in question and everyone else’s practice too. Doing this, the teacher created educational value for everyone.

Residents value various other ways to create efficient discussion. In their view, residents themselves can contribute to efficiency by posing leading questions or raising an issue for discussion. The group should help define the issue if it is still unclear for the resident speaking. These actions focus the discussion onto the main point of value for residents and allows an issue to be generalized from a specific situation to something recognizable to others. To enhance efficiency, teachers should make a list of cases to be discussed at the start of the session. This allows for proper time management and provides clear reasons for cutting short long stories. If the conversation trails off, teachers should turn the focus back on track to the main issue, thus serving the educational end of this particular discussion. The following comment from a residence underscores the importance of this tactic:

Yes, here we’re going back to […] the very practical, um, almost in the direction of giving tips. But just before this [happened], there was this nice interaction where [a resident] said, ‘You know, I’m scared of what others think of me.’ And then I think, yes, but that’s where you [the teacher] can draw the line again. Then I think, ah if only you [the teacher] intervened at this point, we could keep it going and also, I think, go quite a bit deeper. But now a question pulls it from the deep back up to the superficial and then I think oh, what a pity. […] It was going so smoothly just now. […] It’s a shame, that in the group or that a teacher, you know […] I think that if this point were taken up […] then you’d get there much faster, because it can take ages at times (C811).

Though structuring is generally valued for contributing to efficient interaction, it can backfire by cutting short extensive exploration and dynamic detours in unpacking complex cases. Fixed procedures “remove all spontaneity and the learning curve, too” (B859), much like teachers intentionally withholding guidance leaves residents “swimming” (A831) for unseen shores. According to the residents, dynamic structuring nudges interaction efficiently on course toward value for all.

### Collaborative reflection: activities

Residents’ normative orientation to *inclusivity* *and* *diversity*, *safety* and *efficiency* in accomplishing educational value for all is reflected in their perceptions of the value of activities that take place in the various phases of interaction: telling, exploration, discussion, and conclusion. Most attention (in terms of time spent in the recorded sessions and interview time devoted to it) is paid to the discussion phase. Telling and conclusion tend to be short phases, although the telling phase can be extensive if a resident’s aim is to vent whatever is on their mind. The conclusive phase considers all phases relevant to educational uptake.

#### Telling

According to one resident, the potential of the telling phase is determined by the space it is allowed. Telling a story is an interactional accomplishment that requires a longer stretch of talk—ideally uninterrupted. As residents point out, interjections may contribute to efficiency by shortening verbose tellings, but at the same time undermine the functional freedom to take and be given “the space to vent anything and everything you want to share” (B851). Everyone else “shuts up and listens” (G856), withholding questions, opinions, advice, and judgments for later phases, thus constituting inclusivity and safety as the teller proceeds.

For a telling to have educational value, residents point to the importance of the ‘tellability’ and ‘discussability’ of the story. Not all experiences provide ‘tellable’ stories—in the sense that they have a point—and not all tellable stories are ‘discussable’—in the sense that they either open up the grayish floor between guideline-white and unethical-black or induce a stirring of emotion (“at some point, everyone gets triggered here”, C806), betraying the participants’ relation to the issue at hand. Against this norm, bringing up purely medical or procedural questions has limited value for some:

I think we either get to the solution very fast, […] following the guideline, or people have their own opinion and, yeah, they don’t change [that] easily. That sort of stays the way it is (G856).

Yet, stories on straightforward medical topics are sometimes considered tellable for their uniqueness (“most likely, others haven’t come across this either”, A715), which could make them perfect learnables to share with fellow residents. Whatever the topic, therefore, stories become *tellable* and *discussable* for residents whenever the stories address something that carries an urgency or relevance in terms of professional standards and competent practitioner behavior. Discussing that topic would contribute educational value for all the future doctors present.

#### Exploration

Following a resident’s telling, participants usually ask for clarification, probing for additional information or to determine of which ‘type’ this experience is a ‘token’. In residents’ words, clarification helps to understand “how we can best help you” (G856) in the search for answers, recognition, or whatever is expected from this case discussion. In this phase, “directed, continuous attention to uncover the aim” of this telling is valued highly by several residents. As one resident observes, such attention directs the focus in complex stories and contributes to a useful learning uptake for the teller. Residents acknowledge the difficulty and importance of striking a balance between inclusivity/diversity and safety on the one hand, and efficiency on the other. One resident explains,

“The one says this, the other says that, and in a way that’s very positive. It ensures safety, and it’s natural conversation, but to be a bit more constructive and time-efficient, it’d be good if once in a while someone called out, what’s your question?” (B869).

Structure, thus, is considered essential in this phase.

According to several residents, a huge upshot of this phase is the information it gives about how far the teller wants to disclose themselves. Exploration allows the group to “feel out” the teller (G856), while the teller is allowed to set limits. Taking enough time for “edging” toward the possibly emotional core of the issue instead of “going smack bang” into it (G856) can be functional, even if less efficient:

“If you go in directly with ‘what does it do to you?’ then it’s rather confrontational. You may need some kind of detour to get more comfortable in that setting” (C811).

Evidently, efficiency should sometimes be subordinate to safety in this phase.

Residents’ evaluate the variety of exploratory questions that may be asked positively, turning to the importance of diversity for promoting understanding of the issue at hand:

Just like [name of fellow resident], who asked, ‘What [kind of] help does she [the patient] actually want?’ Well, I wasn’t thinking about that at that point. So that again is an eye opener. And now I realize that, yes, wait, in this case the problem is […] (A823).

The posed questions reflect the diversity of perspectives other residents may have: “very many different characters, people who react differently and have different ways of being a GP” (B859). Diverse contributions foster “good dynamics” and stops the group from “spinning its wheels [i.e. wasting time]” (B859), which again shows the residents’ orientation to progress and efficiency.

#### Discussion

Usually, exploration naturally evolves into discussion, a much commented on phase in the interviews. According to the residents the discussion phase is where individual cases should be treated as tokens of a type by transforming the specific issue into a collectively relevant learning issue. One resident reported: “Here we’re all thinking, oh this could happen to me too. What can we learn from this case to prevent it happening?” (A831). Highly valued contributions dive deeper into the issue to suggest potential causes, explore possible directions, and hint at solutions. Residents may share similar stories, which may function positively as a display of recognition and trigger a sense of ‘we’re all in this together’, but can also divert the conversation onto a side-track with no added value. Still, those stories signal the relevance of the discussed issue to another resident, a factor valued as a marker of inclusivity and a clear benchmark of value *for all*.

Teacher participation is regarded as indispensable in the discussion phase. Although too much interference is unwanted, residents expect teachers to monitor the discussion for ‘no go’s’ and to comment on unprofessional behavior. If they do not, one resident explained, “it would be like a GP who’s been in the business for years is approving it [unprofessional behavior]” (A831). Also, residents expect teachers to lead the discussion to topics they know to be important from first-hand experience:

Yes I do expect a teacher… what I really appreciate about these teachers is that they do lean back a lot and let things happen and also trust that we will be able to question each other and get somewhere. Um, but still, he [the teacher] is the hands-on expert. So, at some point I do want to know from him, yes, how does it work or how do you do that? […] Yes, that’s what he’s here for, isn’t he? (C811).

This resident points out two teacher behaviors that enhance educational value in this phase: (1) leave room for the group’s process (which may be less efficient than strictly structured discussion directed straight at the learning issue), and (2) monitor the conversation and jump in with expert knowledge (the voice of experience) when needed. Both behaviors are presented as contributing to the group’s learning process.

#### Conclusion

In this final phase, residents value a teacher’s summary that highlights the ‘learnables’ of the discussion. This builds educational value for all, as it creates an opportunity to “collectively draw a personal note, the lesson from it” and also emphasizes any message of importance for the teller (A845). These summaries may be provisional, not intended to strike the final blow on *the* solution or outcome, but rather to call everyone’s attention to the seeds that have been sown in the attempt to grow toward professional standards. Ideally, *each* resident present—perhaps the teachers as well—would find something *valuable* in each discussion. It could be a concrete solution, but an abstract ‘nudge’ or ‘setting in motion’ with long-term effects is more likely, according to this resident:

She’s been asked so many questions that I assume she’ll have to keep on processing [for a while]. The group doesn’t have to give the answer. With all the questions she’s been asked, she could come across someone, and then she might think, ‘hey, that fits me precisely’ or something. I think we can set things in motion right here, or get things going and let it go on outside [the group]. To put it bluntly, I think it seldom happens… you might be able to use a tip from the group, but things are so personal that to really make it fit, even more so when it concerns very personal things, that almost never happens (C811).

Whatever it may be, then, if you “get something out of it” (D700) either now or in the future, the discussion has proved its merit.

## Discussion

Based on our qualitative analysis of residents’ narratives in reflective video-stimulated interviews, we synthesized shared normative orientations on value in collaborative reflection sessions. Residents describe the potential of collaborative reflection sessions as a concentric construction of educational value for future practice for all. In their views, *inclusivity and, diversity*, *safety* and *efficiency* are necessary for transforming unique experiences into tokens of recognizable issues that are meaningful to discuss in the face of future practice. These norms guide their assessments of specific teacher and resident behavior throughout the case discussion.

Our findings suggest three main features of the collaborative reflection interaction that contribute to educational value for all. First, the collaborative nature of the interaction. The value of *group* interaction resonates with extant reports of narratives of students and residents about the value of collaborative reflection on practice experiences (Chen and Hubinette 2017; Zou et al. 2019). The group setting allows residents to *collaboratively* construct individually relevant ‘learnables’ (Koschmann et al. 1997; Veen and de la Croix 2017) that integrate diverse views on professional practice. The educational potential of such dialogic environments of shared meaning making has been recognized in many educational contexts (see e.g., Mercer and Littleton 2007; Reznitskaya et al. 2009).

A second feature of the interaction that contributes value is *storytelling* as a ‘tool’ to collaboratively reflect during these sessions. Storytelling is the vehicle used to construct the reality of past experiences (Arminen 2004; Bruner 1991; Warmington and McColl 2017), which creates new ways to view the self, others, and the profession (Hardy 2017; Sandars and Murray 2009). The identity work that is done through storytelling makes relevant the discussion of others’ relation to themselves, the situation, and the future profession. Such shared meaning-making promotes the formation of professional identities (Chen and Hubinette 2017; Wald et al. 2015). It forms the machinery, the mechanism, that creates educational value from a single experience. This finding thus reflects the possible effectiveness of narrative pedagogies described in the broader literature (e.g., Brady and Asselin 2016).

The third feature that contributes to value creation during this educational activity is the role of the teacher. As role models, teachers in our study were perceived as a valuable resource and tested benchmark for professional practice. Their expert position brings valued opportunities for pointing out inconsistencies, noticing and dealing with strong emotions, and probing for thought-provoking conversation (Sandars 2009). Also, as moderators, teachers facilitate structured spontaneity (van Braak et al. submitted). Far from creating a dictated environment (Zou et al. 2019), the teachers’ responsibility is to facilitate an open, dialogic environment for learning. Though it may sound counterintuitive, residents in our study stated that clear boundaries and strict procedures create the space for vulnerability, confidentiality and trust (cf. Gallagher et al. 2017). Whatsoever fits these boundaries is likely to contribute to educational value for all.

The current synthesis of GP residents’ normative orientations on value in collaborative reflection sessions develops our understanding of the educational aims of these professionals and their perceptions of ways to realize those aims using a new methodological approach. Two aspects of that approach strengthen the study’s findings. First, during data collection, the interviewers used limited *prompting*. In contrast to elicited responses, responses in our interviews indicate what the residents themselves consider relevant or noteworthy enough to report amid a sea of possible topics and observations that such one-hour recording could raise (van Braak et al. 2018). Also, as responses to recordings of actual interactions, the residents’ comments on value or lack thereof were very *specific* (i.e., “*this* question is valuable at *this* moment, because it contributes to *this* aim”). Both features contribute to a detailed understanding of what is valued and why. Second, the value of our residents’ perspectives on valuable features of collaborative reflection sessions is corroborated by the analytic move to synthesize individual residents’ narratives in underlying shared normative orientations (Maynard and Heritage 2005). The resulting normative orientations on valuable collaborative reflection practices describe the general features of specific activities and behaviors that lend these their value. The general nature of these features makes the findings applicable beyond the specific evaluated situation. Also, their broad character allows teachers to engage with the findings considering their own practice—something a summary abounding in individual residents’ ifs and buts would be unlikely to instigate.

Despite its affordances, however, the methodological approach also has two limitations. First, conducting video-stimulated interviews is time consuming. In addition, it is expensive to hire external interviewers who would be more likely to create a safe environment for criticism than teachers of these sessions would. Therefore, the details of our study’s approach may not suit the limited time and resources available in educational practice. For application of this methodology to improve educational practice, we recommend a ‘light’ version of the approach. Even if just one or two participants would take 10–15 min to reflect on short recordings of education, their reflections would provide rich, empirically related ‘snapshots’ for teachers to respond to. Provided that the residents’ reflections are interpreted for what they really are (subjective, situational interpretations of education), these reflections likely stimulate teachers to (re)think and (re)design educational practices, thus fostering professional teacher development. A second limitation is the impossibility of assessing whether residents’ views on value and lack thereof are justified. Even highly valued teacher interventions may not have accomplished educational value for all. Therefore, we plan to use the findings of our study as the basis for an analysis of the moments in the video that residents evaluated. When we examine what happened in the sessions at those moments, do we find that the action that was evaluated in the interview had particularly negative or positive interactional consequences?

In conclusion, our synthesis of normative orientations displayed in residents’ narratives about valuable features of collaborative reflection shows how participants’ perspectives offer deep and detailed insight into their situational understanding of the local teaching context. Although residents are typically not experts in didactics (Stark and Freishtat 2014), their perceptions are an invaluable resource for understanding “how reflective learning within the curriculum can be better developed to increase engagement from learners” (Vivekananda-Schmidt et al. 2011, p. 1). As such, they form our key to unlock educational value for all.
